# Analysis of Combining SAR and Optical Optimal Parameters to Classify Typhoon-Invasion Lodged Rice: A Case Study Using the Random Forest Method

**DOI:** 10.3390/s20247346

**Published:** 2020-12-21

**Authors:** Jinning Wang, Kun Li, Yun Shao, Fengli Zhang, Zhiyong Wang, Xianyu Guo, Yi Qin, Xiangchen Liu

**Affiliations:** 1 College of Geodesy and Geomatics, Shandong University of Science and Technology, Qingdao 266590, China; wjn2018sk@gmail.com (J.W.); wzywlp@163.com (Z.W.); guoxianyu1@126.com (X.G.); 2 Aerospace Information Research Institute, Chinese Academy of Sciences, Beijing 100094, China; shaoyun@radi.ac.cn (Y.S.); zhangfl@radi.ac.cn (F.Z.); liuxc@radi.ac.cn (X.L.); 3 College of Resources and Environment, University of Chinese Academy of Sciences, Beijing 100049, China; 4 Laboratory of Target Microwave Properties (LAMP), Zhongke Academy of Satellite Application in Deqing (DASA), Deqing 313200, China; 5 School of Earth Science and Resources, China University of Geosciences (Beijing), Beijing 100083, China; qy_cugb@163.com

**Keywords:** sentinel-1, sentinel-2, lodging rice, random forest classification

## Abstract

Lodging, a commonly occurring rice crop disaster, seriously reduces rice quality and production. Monitoring rice lodging after a typhoon event is essential for evaluating yield loss and formulating suitable remedial policies. The availability of Sentinel-1 and Sentinel-2 open-access remote sensing data provides large-scale information with a short revisit time to be freely accessed. Data from these sources have been previously shown to identify lodged crops. In this study, therefore, Sentinel-1 and Sentinel-2 data after a typhoon event were combined to enable monitoring of lodging rice to be quickly undertaken. In this context, the sensitivity of synthetic aperture radar (SAR) features (SF) and spectral indices (SI) extracted from Sentinel-1 and Sentinel-2 to lodged rice were analyzed, and a model was constructed for selecting optimal sensitive parameters for lodging rice (OSPL). OSPL has high sensitivity to lodged rice and strong ability to distinguish lodged rice from healthy rice. After screening, Band 11 (SWIR-1) and Band 12 (SWIR-2) were identified as optimal spectral indices (OSI), and VV, VV + VH and Shannon Entropy were optimal SAR features (OSF). Three classification results of lodging rice were acquired using the Random Forest classification (RFC) method based on OSI, OSF and integrated OSI–OSF stack images, respectively. Results indicate that an overall level of accuracy of 91.29% was achieved with the combination of SAR and optical optimal parameters. The result was 2.91% and 6.05% better than solely using optical or SAR processes, respectively.

## 1. Introduction

Rice is a major food source for nearly half of the world’s population. It is widely planted in China [1], making China the largest global rice producer [2]. Rice lodging is a common disaster which is mainly caused by typhoons or hurricanes. During these events, strong winds bend the rice stems and heavy rain causes flooding of the crop fields, resulting in rice grains and roots to rot after prolonged soaking, having a serious effect on paddy rice production [3]. The magnitude of rice production and grain quality will consequently decrease as photosynthesis processes are disrupted due to self-shading, and translocation of nutrients and carbon from roots to grain is interpreted after lodging [4,5]. It is, therefore, important to quickly and accurately determine the range and location of lodged rice fields for disaster assessment, emergency response and resource allocation.

The traditional method of lodging rice monitoring uses on-site visual observation and assessment, methods which are inefficient and expensive. The rapid development of remote sensing technology has enabled the development of a cost-effective and multi-scale method to monitor lodging crops [6]. As rice lodging is mainly caused by typhoons, which are often accompanied by storms and heavy rain, monitoring studies of lodging rice using remote sensing technology has mainly focused on optical and infrared sensors on unmanned aerial vehicles (UAVs) that operate below cloud cover. Liu et al. [7] used principal component analysis (PCA) and probabilistic neural network (PNN) to demonstrate that healthy and lodged rice can be distinguished using hyperspectral data. Yang et al. [8] combined spectral indices, texture information and the digital surface model (DSM) extracted from UAV images to acquire high classification accuracy of lodging rice using a decision tree method. Liu et al. [9] constructed a model to recognize lodged rice using support vector machine (SVM) and particle swarm optimization (PSO) by analyzing temperature, color and texture information of lodged rice collected from UAV visible and thermal infrared images. Although UAV plays an important role in monitoring rice lodging, the imaging width of UAV is limited to the local or experimental scale; therefore, it cannot meet the requirement of rapid regional-scale monitoring [10].

As synthetic aperture radar (SAR) microwaves can penetrate clouds, haze and light rain, SAR backscattering signals obtained from radar satellites are not generally influenced by weather conditions. SAR data are rapidly collected and can cover areas. SAR signals are also sensitive to the morphological structure of rice canopies [11,12]. Radarsat-2 and Sentinel-1 data have been explored to monitor lodging crops. Previous investigations have shown that multiple polarimetric features have the ability to distinguish between lodging wheat and sugarcane from healthy crops based on Randarsat-2 fully polarimetric data [6,13,14]. By analyzing the sensitivity of polarimetric features extracted from Sentinel-1 to lodged corn, Han and Shu et al. [15,16] constructed a model to classify lodged corn with different lodging angles. Chauhan et al. [17,18] explored the ability of Radarsat-2 and Sentinel-1 data with multiple incidence angles to classify healthy wheat and lodged wheat with different crop angles of inclination (CAI). They found that images with low incidence had good performance for high CAI wheat identification, and vice versa.

Although satellite-based optical images are often contaminated by cloud shadows [19], it has been demonstrated that multi-spectral indices of optical data are sensitive to lodging crops [20]; optical and SAR data provide more information for distinguishing and monitoring lodging crops. Although the information synergy of SAR features and optical indices has been widely studied in crop variety identification and crop mapping [1,19,21], few applications for lodging crop mapping have been undertaken. Having the characteristics of a fast-revisiting period, a wide-coverage area and free-access, Sentinel-1/2 are suited to efficiently monitor lodging rice after a typhoon or hurricane [22]. Wu et al. [23] proposed a workflow for mapping lodging paddy rice area based on Sentinel-1 and Sentinel-2 data in the Google Earth Engine (GEE) platform; however, limitation parameters (VV, VH, NDVI and EVI) were used and analyzed, and the single parameter image was used in the lodging rice classification step.

In this study, 10 Radar features of Sentinel-1 and 10 spectral indices of Sentinel-2 were evaluated for lodging rice sensitivity, and a selection model was developed for acquiring the optimal sensitivity parameters for lodging rice (OSPL). Optimal SAR features (OSF) and optimal spectral indices (OSI) were then selected based on the OSPL model. Three classification results were acquired using the Random Forest (RF) classification method with the input of OSF, OSI and integrated OSF–OSI. The accuracy of the three classification results was evaluated using the validation data and confusion matrix.

## 2. Study Area and Data

### 2.1. Study Area

**Figure 1:**
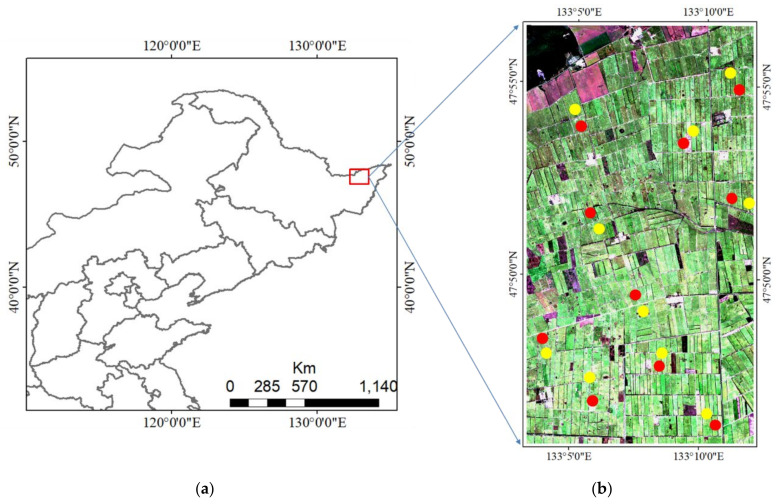
The study area (**a**) and location of the sample fields overlaid on a Sentinel-2 true color synthesis image (**b**) acquired on 17 September 2019. Yellow circles indicate healthy rice plots and red circles indicate lodge plots.

The study area is situated within Qindeli farm (Figure 1), Tongjiang City, Heilongjiang Province, China (47°51′31″ N, 133°7′32″ E). The study area is located in a humid monsoon climate zone, in the third accumulated temperature zone in Heilongjiang Province, being one of the important commodity grain production bases in China. The total cultivated area of Qindeli Farm is about 52,800 hectares, of which rice planting accounts for more than 80% of the total cultivated area. Single season rice is cultivated in the test area, having a growing period from early May to late September.

**Figure 2:**
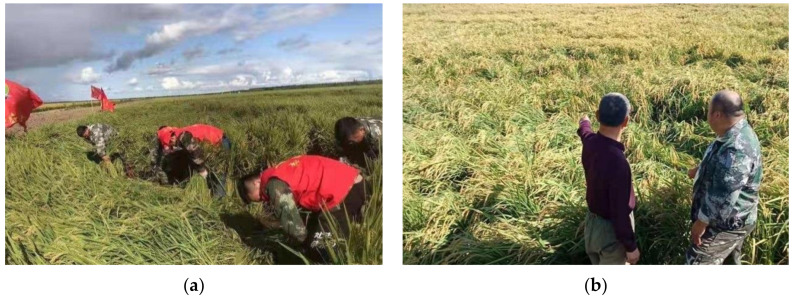
Photos of lodged rice (**a**) and (**b**) in the SanJiang Plain, taken after invasion by typhoon Lingling.

On 7 September 2019, typhoon “Lingling” passed over this area, resulting in heavy rainfall that lasted for two days. During this event, a large area of rice plants (the majority of which were in the mature stage) were lodged, as can be seen in Figure 2a,b.

### 2.2. Experimental Data

In order to explore the sensitivity of different SAR features and spectral indices to lodged rice, three Sentinel-1B and two Sentinel-2(A/B) images over the study area were downloaded from the Copernicus Open Access Hub of European Space Agency (ESA) before and after Typhoon Lingling; the main parameters of Sentinel-1/2 data used in this study were detailed in (Table 1). A single look complex (SLC) format of Sentinel-1 data was used to extract backscatter coefficients of different polarizations and H/A/Alpha polarization decomposition parameters.

Two scenes of Sentinel-2 Level-2A optical images in the UTM/WGS84 projection were cloudless over the study area, and their reflectance was extracted, except for bands 1, 9 and 10, which are irrelevant for crop studies. At the same time, wind speed and rainfall hourly data were collected from the China Meteorological Data Service Center (CMDC) to aid interpretation of results.

Due to the large scale of lodged rice, ground data provided by local farm worker could not provide field scale verification. Therefore, a Pleiades satellite 0.5 panchromatic high spatial resolution image captured on 14 September (within one week of the typhoon event) was introduced to downscale ground data from a local scale to a field scale. In addition, the SRTM (Shuttle Radar Topography Mission) 30 m resolution digital elevation model (DEM) was used for geocoding of Sentinel-1 SAR imagery.

## 3. Methods

### 3.1. Pre-Processing of Remote Sensing Images

Backscatter coefficients from SLC format data of Sentinel-1 were extracted using SNAP 6.0. After applying the orbit file correction, the thermal noise removal and radiometric calibration operator were conducted so that pixel values were directly related to the radar backscatter of the scene. The refined Lee speckle filter was then applied with a 3 × 3 window to eliminate the inherent speckles of the SAR image, and the terrain correction operator was performed so that geometric representation of the image was as close as possible to the real world. Finally, the value of resulting images was converted to dB from linear ($\sigma_{\mathrm{VV}}^{0}$ and $\sigma_{\mathrm{VH}}^{0}$). $\sigma_{\mathrm{VV}+\mathrm{VH}}^{0}$,$\sigma_{\mathrm{VV}-\mathrm{VH}}^{0}$ and $\sigma_{\mathrm{VH}/\mathrm{VV}}^{0}$ were also calculated using the band math operator.

In relation to polarimetric decomposition parameter extraction, IW1 subswath was initially extracted using PolSARpro 6.0. Multi-looking (Azimuth: 1, Range: 4) was then performed followed by C2 matrix extraction. SNAP software assisted in completing part of the data preprocessing, including TOPSAR spilt, Apply Orbit File, Complex Calibration and Deburst. After geocoding and refined Lee filter, a total of five polarimetric features were acquired using H/A/Alpha polarimetric decomposition, including Alpha, Entropy Anisotropy, Span and Shannon Entropy. Different from fully polarimetric data, H/A/Alpha decomposition parameters of dual-pol data were generated from C2 matrix, as:

(1) $$Alpha=\frac{1}{\lambda_{1}+\lambda_{2}}\sum_{i=1}^{2}\lambda_{i}\alpha_{i}$$

(2) $$Entropy=-\sum_{i=1}^{2}P_{i}\log_{2}(P_{i})$$

(3) $$Anistropy=\frac{\lambda_{1}-\lambda_{2}}{\lambda_{1}+\lambda_{2}}$$

(4) $$S\mathrm{pan}={\left|C_{11}\right|}^{2}+2{\left|C_{12}\right|}^{2}+{\left|C_{22}\right|}^{2}$$

(5) $$Shannon=2\log(\frac{\pi eTr\left[C2\right]}{2})+\log(4\frac{\det[C2]}{Tr{\left[C2\right]}^{2}})$$

The process of Sentinel-2 L2A products mainly includes bottom-of-atmosphere (BOA) corrected reflectance, and images were subset to reduce processing time. Finally, the Sen2Res tool was used to improve the spatial resolution of Sentinel-2 products to 10 m resolution whilst simultaneously preserving their reflectance.

### 3.2. Screening of Optimal Sensitive Parameter for Lodging Rice

#### 3.2.1. Statistical Analysis

Ten lodged (L1–L10) and ten healthy (H1–H10) rice sample plots were selected in the study area based on downscaling field data (Figure 1). Inhomogeneity in each plot was eliminated by extracting mean values of SAR features (SF) and spectral indices (SI) (see Section 3.1 for data pre-processing). Sensitivity differences of SF and SI to lodged rice and the reasons for these differences were then analyzed.

#### 3.2.2. OSPL Screening Model

After undertaking sensitivity analysis of SF and SI to lodged rice, an OSPL screening model was developed. Parameters selected by the OSPL screening model were highly sensitive to lodged rice, having a strong ability to distinguish lodged rice from healthy rice. Selection of the OSPL screening model included three important steps:

(i) A sensitivity criterion to rice lodging, γ, was proposed to select parameters sensitive to lodged rice, as:

(6) $$\gamma =\left|\frac{LB_{\mathrm{av}}-LA_{\mathrm{av}}}{LB_{\mathrm{av}}+LA_{\mathrm{av}}}\right|-1.5\times \left|\frac{HB_{\mathrm{av}}-HA_{\mathrm{av}}}{HB_{\mathrm{av}}+HA_{\mathrm{av}}}\right|$$

where $LB_{\mathrm{av}}$ ($LB_{\mathrm{av}}=(L1+L2+\ldots +L10)/10$) is the average value of ten lodged rice plots before the typhoon event; $LA_{\mathrm{av}}$ ($LA_{\mathrm{av}}=(L1+L2+\ldots +L10)/10$) is the average value of ten lodged rice plots after the typhoon event; $HB_{\mathrm{av}}$ ($HB_{\mathrm{av}}=(H_{1}+H_{2}+\ldots +H_{10})/10$) is the average value of ten healthy rice plots before the typhoon event and $HA_{\mathrm{av}}$ ($HA_{\mathrm{av}}=(H_{1}+H_{2}+\ldots +H_{10})/10$) is the average value of ten healthy rice plots after the typhoon event.

When γ is greater than 0, the variation of the corresponding parameter value of the lodged rice plot before and after typhoon is much greater than that of the healthy rice plot. Therefore, parameters meeting the criterion that γ is greater than 0 are sensitive to lodged rice.

It is worth noting that each element in the formula of sensitivity criterion γ is the average value of ten rice plots. As accuracy and reliability of γ value determination will be reduced by the presence of extreme noise values, it was therefore important to evaluate the value of parameters screened in the first step for each plot in the following steps.

(ii) Two parameters (β and ε) were constructed to analyze the variation tendency of SF and SI of all the lodged rice sample plots after lodging, as:

(7) $$\varepsilon_{i}=LA_{i}-LB_{i}(i=1,2,\ldots ,10)$$

(8) $$\beta =\mathrm{sig}n(\varepsilon_{i})(5\leq \beta \leq 10)$$

where, $\varepsilon_{i}$ is the difference between parameter values $LB_{i}$(lodged sample plot before the typhoon) and $LA_{i}$ (lodged sample plot after the typhoon); β is the number of the sign of $\varepsilon_{i}$ with a higher frequency (ε has six positive signs and four negative signs, then β = 6). 

When β is ≥9, the corresponding parameter will be selected. After this step, sensitivity of the selected parameters to lodged rice has good consistency.

(iii) Parameters that fulfill the following criteria were used to distinguish lodged rice from healthy rice.

(9) $$\left(\mathrm{if}\;HA_{\mathrm{av}}>LA_{\mathrm{av}}\right),\;HA_{1/4}^{b}>LA_{\mathrm{Max}}\;\mathrm{and}\;LA_{1/4}^{u}<HA_{\mathrm{Min}}\;\left(\mathrm{if}\;HA_{\mathrm{av}}<LA_{\mathrm{av}}\right),\;LA_{1/4}^{b}>HA_{\mathrm{Max}}\;\mathrm{and}\;HA_{1/4}^{u}<LA_{\mathrm{Min}}$$

where, $HA_{1/4}^{b}$ and $HA_{1/4}^{u}$ are the lower and upper quantile values of healthy rice sample plots, respectively; $LA_{1/4}^{b}$ and $LA_{1/4}^{u}$ are the lower and upper quantile values of lodged rice sample plots, respectively; $HA_{\mathrm{Min}}$ and $HA_{\mathrm{Max}}$ are the minimum and maximum values of healthy rice sample plots, respectively; $LA_{\mathrm{Min}}$ and $LA_{\mathrm{Max}}$ are the minimum and maximum values of lodged rice sample plots, respectively.

### 3.3. Classification of Lodging and Healthy Rice

Before lodged rice and healthy rice fields were classified, interference due to buildings, water bodies, woods or other crops to lodged rice mapping were eliminated using the maximum likelihood classification (MLC) method [24] to extract the rice region as a rice mask shapefile using Sentinel-2 images. The extracted rice mask shapefile was then applied to all OSPL images to remove non-rice regions, and a Random Forest (RF) classifier was introduced to lodged rice mapping, having three inputs of OSF, OSI and integrated OSF–OSI stack images.

**Figure 3:**
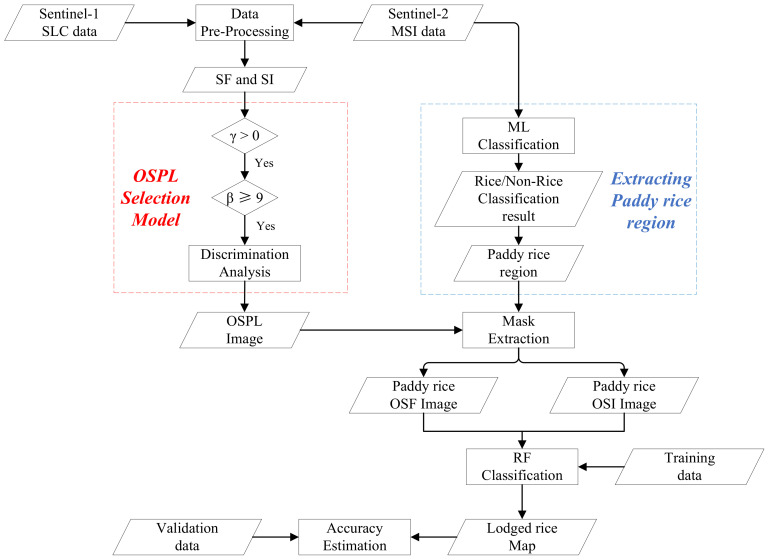
Flowchart of lodged rice mapping in this study.

RF, a supervised machine learning method, is built on Classification and Regression Trees (CARTs) using the GINI index as the calculation principle. This method was developed by Breiam [25] and it has been widely applied to classify crops at different scales [21,26,27,28]. The classification result is generated from ensemble voting results of multi trees for the most popular class. Each tree is trained using sub-sample dataset grabbed from training dataset by bootstrap sampling, and the proportional randomly selected features from the feature dataset are used at each tree node. By excluding the 10 + 10 sample fields previously mentioned, a total of 4303 additional sample point pixels were randomly selected on the basis of downscaling field data and high-resolution Google Earth imagery, of which 2876 pixels were assigned for training, and 1427 pixels were set as a validation dataset. After being fine-tuned numerous times, the RF optimizer had 100 trees, two maximum features and a 0.2 min impurity. Finally, the classification accuracies were evaluated using the validation dataset and confusion matric. The flowchart of the detailed processes for lodged rice mapping is shown in Figure 3.

## 4. Results and Discussions

### 4.1. SAR Parameter Analysis

**Figure 4:**
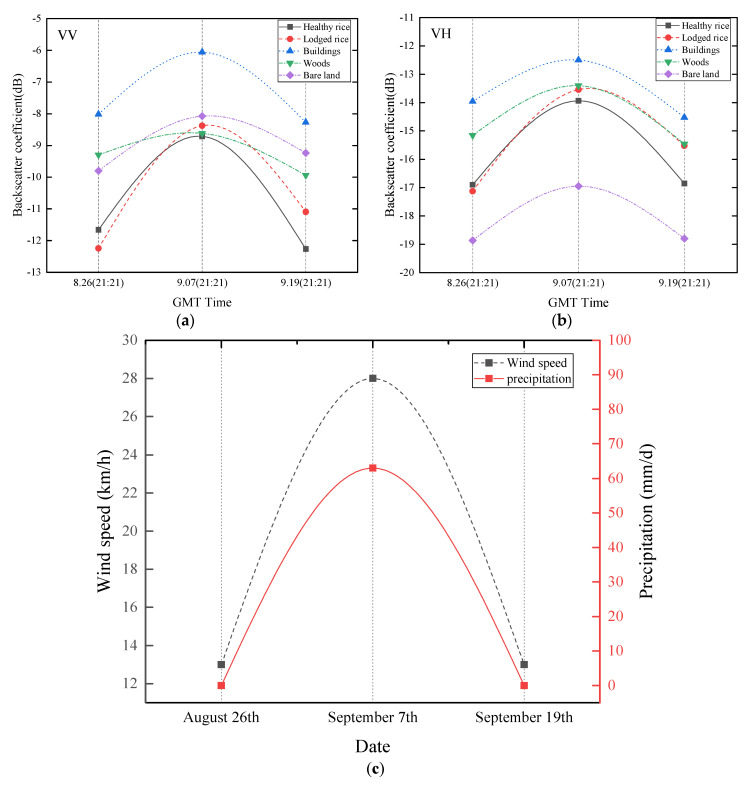
Backscatter coefficients on 26 August, 7 September and 19 September for (**a**) VV polarized and (**b**) VH polarized for rice fields, buildings, woods, and bare land. Corresponding wind speed and precipitation results are shown in (**c**).

The backscatter behavior of rice and other typical features (buildings, woods and barren land) were analyzed before, during and after the typhoon. Backscatter coefficients of VV and VH polarizations (Figure 4a,b, respectively) before (26 August), during (7 September) and after the typhoon (19 September) indicate that rice and other typical features had similar backscatter behavior. This behavior initially increased (26 August to 7 September) before decreasing (19 September). The significant increase in backscatter coefficients on 7 September was mainly caused by heavy rain (63 mm/d, Figure 4c), similar to findings from previous studies [29,30].

**Figure 5:**
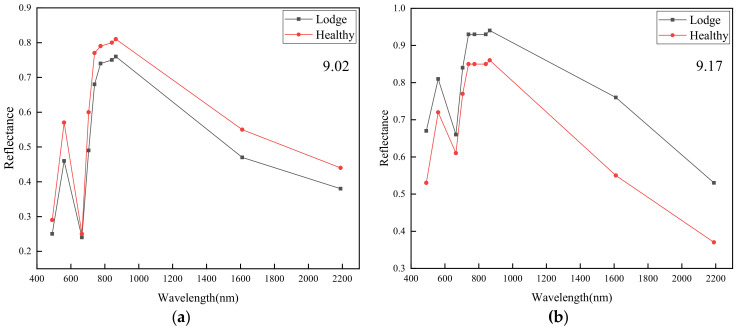
Average spectral variation of lodging and healthy rice on 2 September (**a**) and 17 September (**b**).

The significant increase in backscatter results recorded on 7 September associated to heavy rain, resulted in rice lodging to be overwhelmed. Therefore, images on 26 August and 19 September were only used to explore the capability of SAR in rice lodging mapping. Backscatter results (Figure 5a,b) indicate that backscatter coefficients of lodged rice sample plots (LS) significantly increased from 26 August to 19 September at VV and VH polarizations, and slight difference was recorded from 26 August to 19 September in healthy rice sample plots (HS).

Backscatter coefficients of $\sigma_{\mathrm{VV}}^{0}$ and $\sigma_{\mathrm{VH}}^{0}$ in LS increased by 1.15 dB and 1.61 dB after the typhoon, respectively (Table 2). The increase of $\sigma_{\mathrm{VV}}^{0}$ backscatter coefficients was probably due to a decrease of signal attenuation, associated to the destruction of the vertical structure of the rice canopy during rice lodging [31]; the increase of $\sigma_{\mathrm{VH}}^{0}$ may be the enhancement of multiple scatterings [6]. 

In addition, the increment of $\sigma_{\mathrm{VH}}^{0}$ was greater than $\sigma_{\mathrm{VV}}^{0}$ for LS (Table 2). Two probable factors can account for this phenomenon: (i) When VV polarized wave interacts with rice plants, double scatter is the main component of the total backscatter signals [32], however, as the angle between rice stems and the ground surface decreases after lodging, backscatter signals weaken. (ii) Volume scatter, the main component of total backscatter signals for $\sigma_{\mathrm{VH}}^{0}$, increases due to enhancement of multi scattering after lodging [6].

The polarimetric decomposition parameters, such as entropy (H), anisotropy (A) and average scattering angle (α) of healthy and lodged rice, were also analyzed. H, A and α recorded a low sensitivity to rice lodging, with $\sigma^{L}$ change values of 0.09, 0.02 and 0.02 after rice lodging, respectively (Table 2).

H/A/Alpha, reflecting target scatter mechanisms, have been shown to be strongly sensitive to lodged crops when generated from fully polarimetric data [6,17]. However, the same parameters from dual-pol data were reported by Chauhan et al. [17] to have a lack of sensitivity to lodged crops. This is probably the reason why H/A/Alpha were not sensitive to rice lodging in this study.

### 4.2. Reflectance Analysis

LS and HS spectral behavior were analyzed before (2 September) and after (17 September) the typhoon event (Figure 5a,b). Although rice lodging had not yet occurred, LS and HS reflectance differences in the majority of spectral bands were recorded before the typhoon occurred (Figure 5a), with variety differences between non-resistant and resistant lodging probably accounting for this situation. Due to canopy architecture and biophysical factors (such as chlorophyll and LAI (Leaf area index)) of rice changing after rice lodging, LS reflectance significantly increased in all spectral bands after the typhoon event (Figure 5b) [33,34].

**Figure 6:**
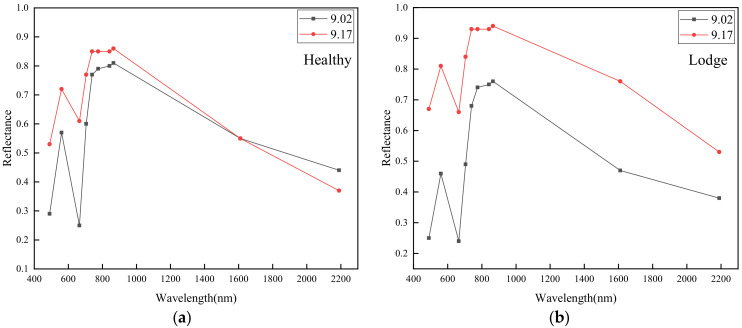
Average spectral variation on 2 September and 17 September for (**a**) healthy rice and (**b**) lodged rice.

In addition, different spectral band reflectance values for each sample plot before and after the typhoon were analyzed (Figure 6a,b). Results indicate that HS reflectance values in almost all spectral bands also increased after the typhoon (Figure 6a), probably indicating a stronger illumination in the study area on 17 September compared to 2 September. Finally, by combining the above conditions of canopy architecture, biophysical changes and illumination difference, the biggest reflectance difference was recorded on 17 September (Figure 6b), recording the greatest contrast in LS reflectance before and after the typhoon.

Results from this analysis indicate that the increase in the magnitude of reflectance values within the VIS–NIR–SWIR region for LS after the typhoon was evident (Figure 6b); reasons for this result have been previously investigated by Chauhan et al. [20]. The reduction in rice chlorophyll content due to disrupted photosynthesis is a major factor increasing reflectance values in the VIS region [5]. Reflectance increases in NIR and SWIR are associated with canopy structure and plant water content changes.

### 4.3. Selecting of OSPL

OSPL screening model incorporating γ and β are shown in Table 3 (for SF) and Table 4 (for SI). According to the criterion of γ > 0, VV, VH, VV + VH, VV − VH, Shannon Entropy, and Span were selected as SAR features with parameters sensitive to lodged rice. As the magnitude of β was highly correlated with positive and negative γ values (Table 3), the ability of γ to determine whether SF (SAR feature) is sensitive to lodged rice was also confirmed.

**Figure 7:**
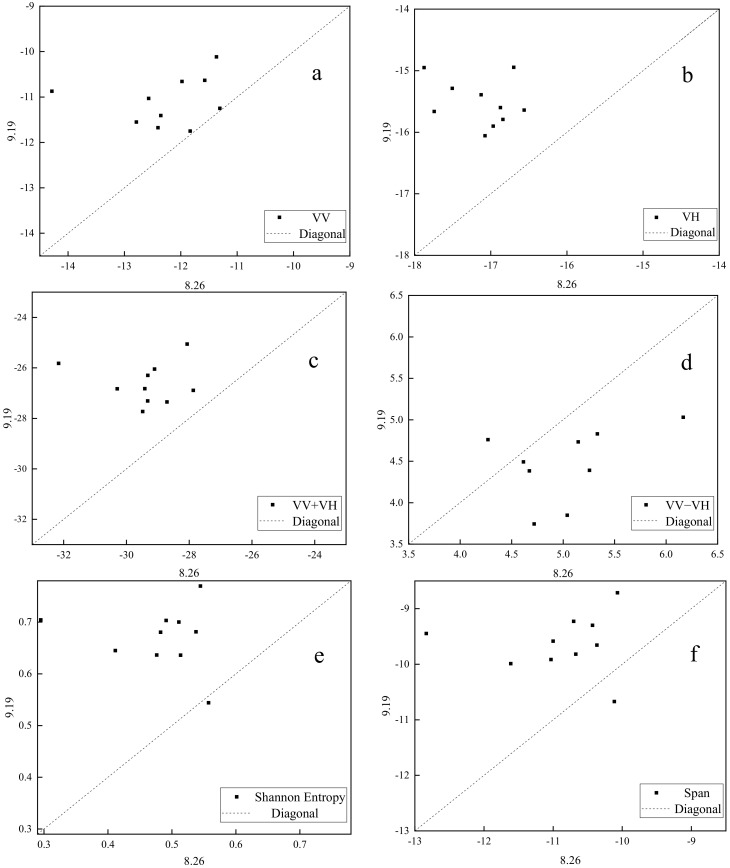
Change trend of lodging rice for (**a**) VV, (**b**) VH, (**c**) VV + VH, (**d**) VV − VH, (**e**) Shannon Entropy, (**f**) Span before and after lodging.

Correlation scatterplots of SF screened by γ were constructed and each scatterplot included ten points (Figure 7). As the X and Y coordinate values of each point denotes SF values of one lodged sample plot before and after the typhoon, respectively, the distribution of these points reflects the consistency of SF changes after rice lodging. Here, VV, VH, VV + VH, VV − VH, Shannon Entropy and Span values changed after rice lodging, recording good consistency (scatter points are concentrated on the onside of line 1:1) (Figure 7). The value of β also indicated the consistency of parameter changes after rice lodging.

With regard to SI, based on the criterion of γ > 0 (Table 4), all spectral bands apart for Band 4 (Red) were selected as sensitive parameters to rice lodging, indicating that the majority of the band indices of Sentinel-2 are sensitive to lodged rice. A similar conclusion was recorded in a study mapping lodged wheat [20]. However, different from SF, β and γ for SI did not have a strong consistency for judging rice lodging sensitivity as, except for Band 3 (Green), β values for all other spectral bands reached 10 (including Band4). The higher β values of SI, therefore, were not credible because differences in illumination between 2 and 17 September interfered with results (see Section 4.2).

A total of 14 parameters were selected using the first and second steps of the OSPL screening model, including six SF (VV, VH, VV + VH, VV − VH, Shannon Entropy and Span) and eight SI (VIS-Blue (Band 2), Vegetation Red Edge (Bands 5, 6, 7 and 8A), NIR (Band 8) and SWIR (Bands 11 and 12)). Parameters with good performance for distinguishing healthy and lodged rice (detailed information is provided in Section 3.2.2) were obtained using the third step. Finally, VH, VV + VH, Shannon Entropy, SWIR1 (Band 11) and SWIR2 (Band 12) were selected as optimal sensitive parameters to rice lodging using the OSPL screening model.

**Figure 8:**
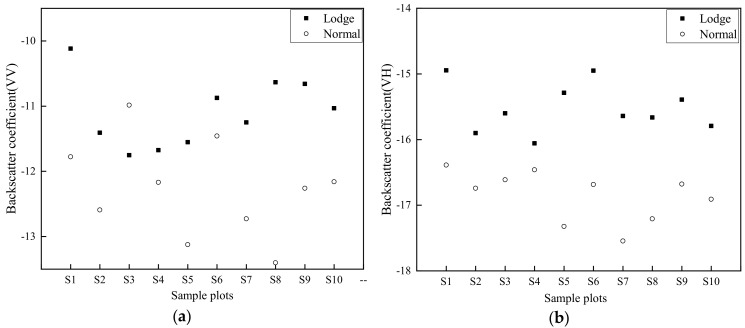

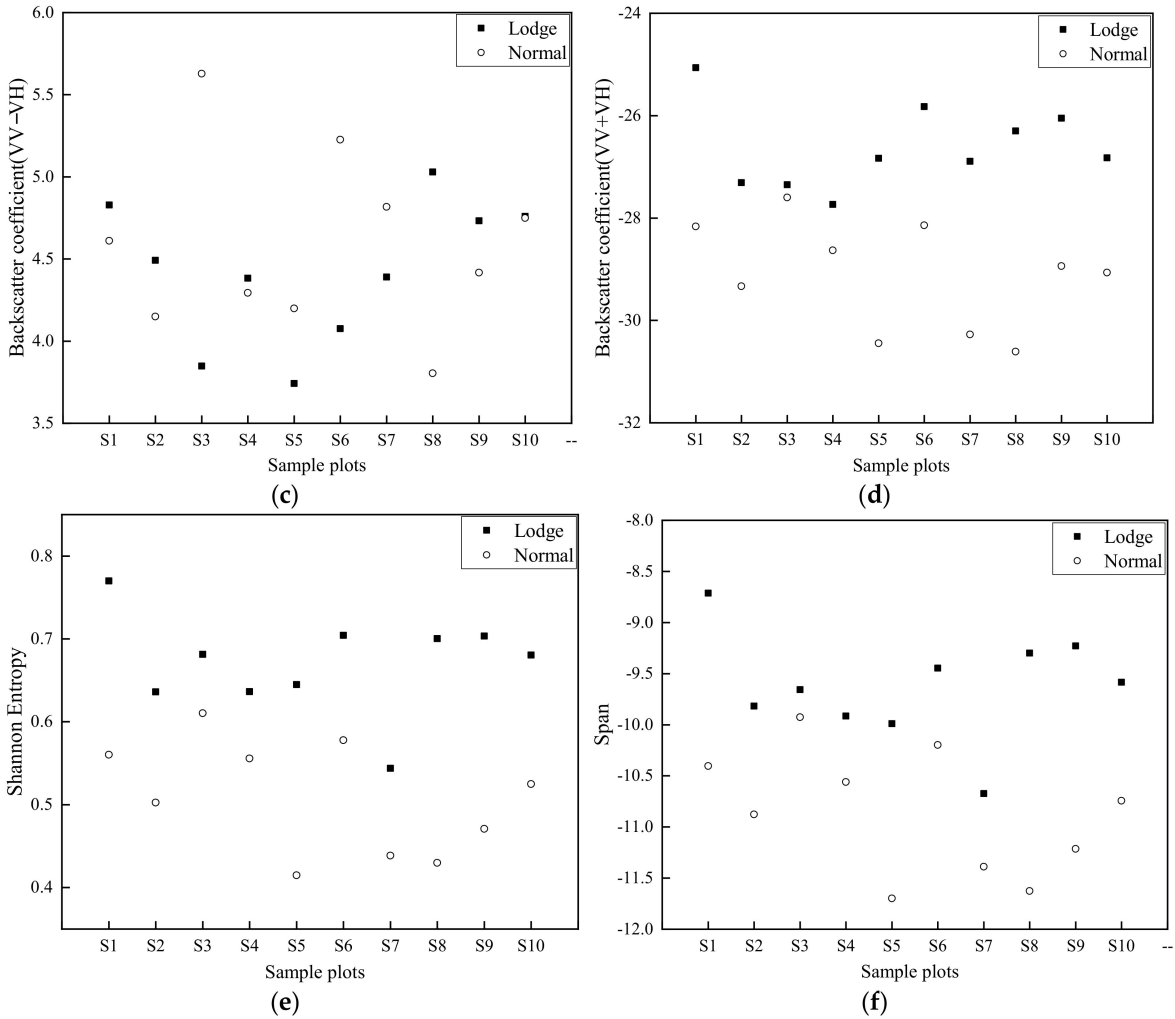
Discrimination results of Sentinel-1 SAR features after the typhoon (19 September). (**a**) $\sigma_{\mathrm{VV}}^{0}$; (**b**) $\sigma_{\mathrm{VH}}^{0}$; (**c**) $\sigma_{\mathrm{VV}-\mathrm{VH}}^{0}$; (**d**) $\sigma_{\mathrm{VV}+\mathrm{VH}}^{0}$; (**e**) Shannon Entropy; (**f**) Span.

**Figure 9:**
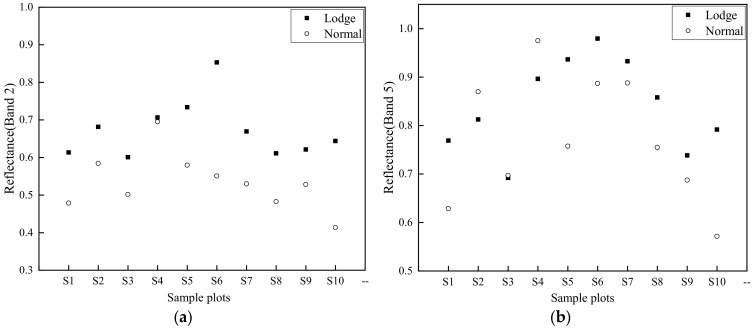

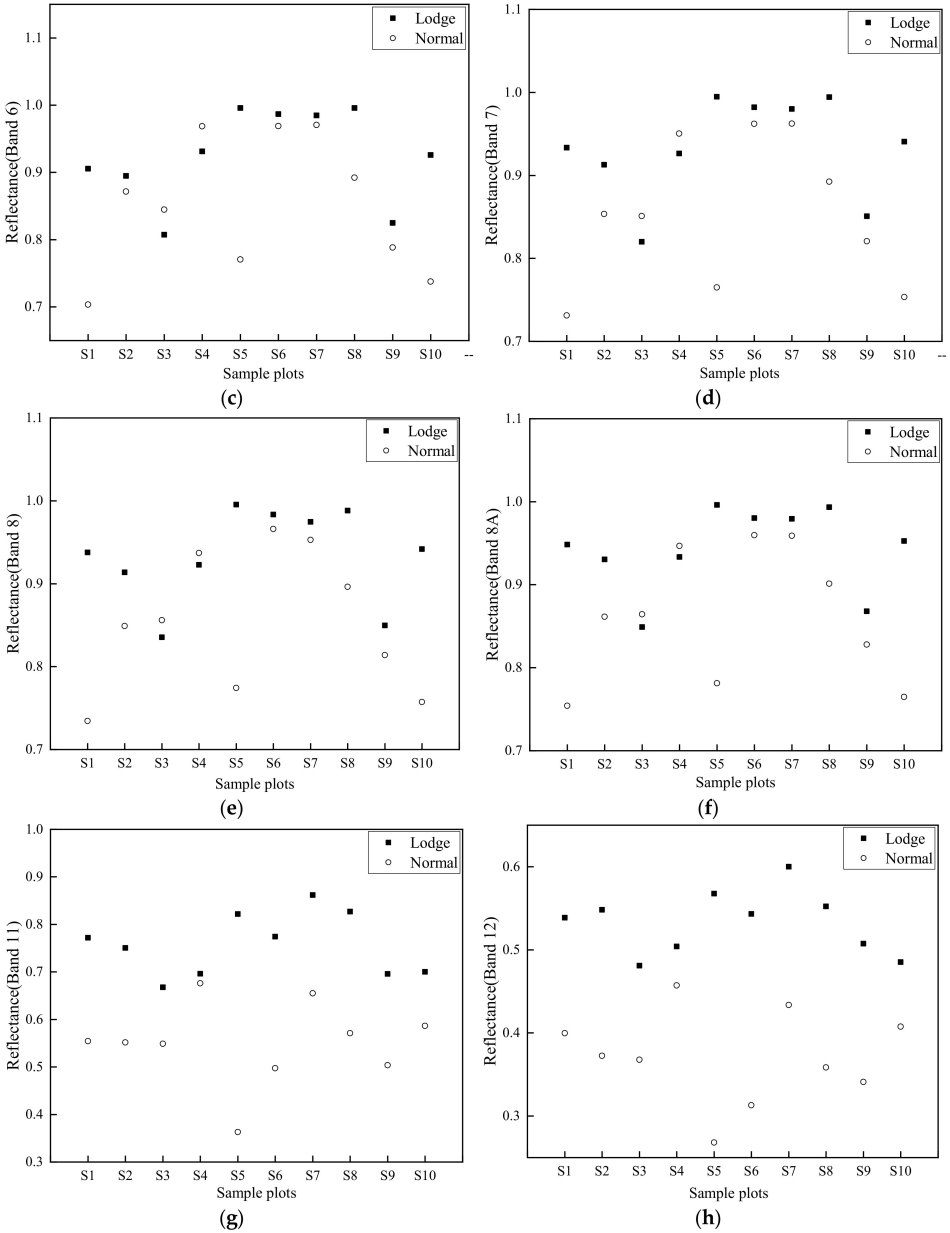
Discrimination results of Sentinel-2 spectral indices after the typhoon (17 September). (**a**) Band 2 (Blue); (**b**) Band 5 (Red Edge 1); (**c**) Band 6 (Red Edge 2); (**d**) Band 7 (Red Edge 3); (**e**) Band 8 (NIR); (**f**) Band 8A (Red Edge 4); (**g**) Band 11 (SWIR1); (**h**) Band 12 (SWIR2).

Discrimination scatter plots of all sample plots (S1-S10) for SF and SI (Figure 8 and Figure 9) were selected using screening steps 1 and 2, respectively. Results indicate that all selected OSPLs had almost no overlap between healthy and lodged sample rice plots, indicating that OSPL has sufficient ability to distinguish lodged rice from healthy rice.

In this study, $\sigma_{\mathrm{VV}}^{0}$ and $\sigma_{\mathrm{VH}}^{0}$ were found to be sensitive to lodged rice (see Section 4.1). Similar conclusions were recorded when the C-band spaceborne SAR was used to monitor wheat and corn lodging [16,18,20]. Han et al. also used VH and VV + VH as optimal polarization parameters to classify lodged corn with a different lodging degree [15].

### 4.4. Mapping Healthy and Lodged Rice Based on RF and OSPL

**Figure 10:**
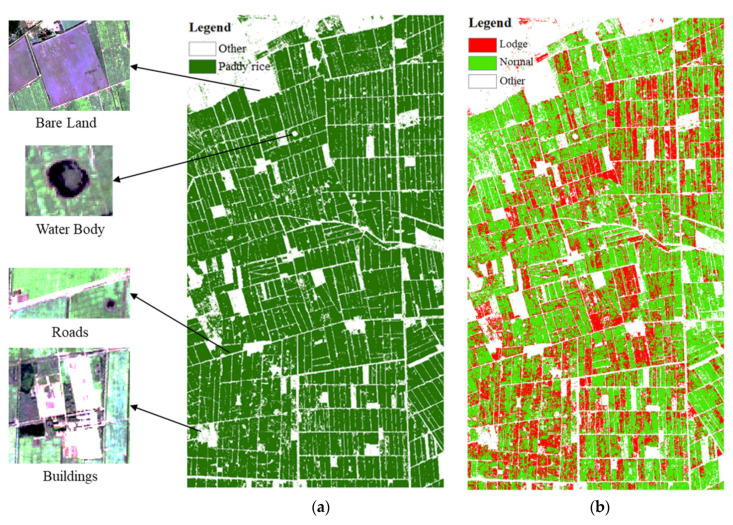
(**a**) Shape of rice region and detailed classification; (**b**) the RF classification result using the integrated OSF–OSI stack image.

Rice region extraction was conducted using optical images and MLC (Figure 10a). The study area contained a total of 2,187,630 pixels with 10 m space resolution, of which 1,754,260 pixels (80.19%) were paddy rice and the remaining 433,370 pixels (19.81%) were non-rice region (Figure 10a). The recognition ability of MLC to various and small features was identified using the detailed classification features of bare land, water body, roads and buildings (Figure 10a). The shape of the paddy rice region was used to eliminate the non-rice region of OSPL image, and lodged rice mapping was undertaken using a fine-tuned RF classifier (Section 3.3) according to OSFs and OSIs selected by the OSPL selection model. Three cases were designed to evaluate the capability of SAR and optical data in lodged rice mapping. Case I: Optimal SAR features were used for classification; Case II: Optimal optical spectral indices were used as the inputs of classification; Case III: The integration of optimal SAR and optical parameters were used as the inputs of classification. Accuracy results of lodged rice mapping using the three cases are shown in Table 5.

Producer’s accuracy (PA), user’s accuracy (UA), overall accuracy (OA) and Kappa coefficient of confusion matric were calculated based on classification results and validation data. PA values represent the pixel number of correctly classified in one category divided by the total pixel number in the corresponding category of ground truth data. UA values were computed by dividing the pixel number of correctly classified in one category by the total number of classified in the corresponding category. OA values are the result of correctly classified pixels in all categories divided by the total pixel number of all categories.

Through the observation and comparison for intersection pixel distribution of ground truth (GT) and classification category (CC) and values of PA and UA, we found that commission error of lodge class and omission error of healthy class were larger in Case I, while commission error of healthy class and omission error of lodge class were larger in Case II. Finally, the commission and omission errors for healthy and lodge classes were reduced by combining SAR and optical information with mutually complement characteristics, and the values of OA and kappa coefficient also increased in Case III compared to Case I and II. The integrated OSF–OSI classification result image (Figure 10b) indicates lodging rice (red region), healthy rice (green regions) and non-rice regions (white regions). In Case III, lodging rice accounted for 34.63% of the overall paddy rice region, being concentrated in the Northeast, Northwest and Central regions in study area (Figure 10b).

This method used free-to-download Sentinel-1/2 spaceborne data and a whole set of processing flow paths executed in available software, and RF classification was employed based on fewer adjusted parameters. However, interferences of cloud and mist to Sentinel-2 optical data and heavy rain to Sentinel-1 SAR data limit the applicability of this method.

## 5. Conclusions

In this study, multiple SAR and optical parameters extracted from Sentinel-1/2 were analyzed for lodged rice sensitivity. An OSPL selection model was developed to select optimal parameters for lodged rice mapping, and five parameters were selected based on OSPL screening model, of which three parameters were OSF (VH, VV + VH and Shannon Entropy) and the remaining were OSI (Band 11 and Band 12). Finally, a higher classification accuracy was achieved using ORF–OSI combination images based on fine-tuned RF classifier. Some of the findings are as follows:

(i) The VV and VH polarization signals of Sentinel-1 C-band data might be significantly enhanced under heavy storms in farmland regions; (ii) alpha (α), entropy (H) and anisotropy (A) of H/A/Alpha polarimetric decomposition based on dual-polarized data have a low level of sensitivity to lodged rice; (iii) VV and VH of Sentinel-1 are largely sensitive to lodging rice due to structural changes of canopy and stalks. The majority of spectral bands were sensitive to lodging rice mainly due to biophysical/biochemical and rice canopy architecture changes.

The limitations of this work and areas for improvement in future investigations have the following points: (i) In this study, the time interval between the typhoon transit time and acquisition time of Sentinel-1 and Sentinel-2 image data was long; available data recorded closer to typhoon events should be collected and analyzed. (ii) Large scale lodged rice mapping was not undertaken as lack of the appropriate field data and Google Earth imagery; the classification effects of OSF, OSI and OSF–OSI using RFs on regional-scale can be executed further. (iii) The dual polarized information (VV and VH) used in this study has limitations for exploring the radar polarimetric response of lodging rice; fully polarimetric spaceborne data should be explored in the future as it not only has richer polarization information, it can also provide more variety of polarimetric decomposition information.

## Figures and Tables

**Table 1:** Sentinel-1 and Sentinel-2 data parameters used in this study.

Satellite	Sentinel-2	Sentinel-1
Spatial Resolution	10 m	15 m
Spectrum range	B2, B3, B4, B5, B6, B7, B8, B8A B11, B12	
Polarization mode		VV, VH
Data level	Level-2A	Level-1
Image mode	MSI	IW
Swath width	290 Km	250 Km
Revisit	5d	12d
Acquisition	2 and 17 September 2019	26 August/7 and 19 September 2019

**Table 2:** SF and SI statistical data. $LB_{av}$, $LA_{av}$, $HB_{av}$ and $HA_{av}$ are detailed in Section 3.2.2 (i); $\sigma^{L}$: The absolute value of difference between $LB_{av}$ and $LA_{av}$; $\sigma^{H}$: The absolute value of difference between $HB_{av}$ and $HA_{av}$.

Parameter	$LB_{\mathrm{av}}$	$LA_{\mathrm{av}}$	$\sigma^{L}$	$HB_{\mathrm{av}}$	$HA_{\mathrm{av}}$	$\sigma^{H}$
VV	−12.25	−11.1	1.15	−11.66	−12.27	0.61
VH	−17.13	−15.52	1.61	−16.91	−16.86	0.05
VV + VH	−29.37	−26.82	2.55	−28.56	−29.12	0.56
VV − VH	5.18	4.43	0.75	5.06	4.59	0.47
VH/VV	1.41	1.40	0.01	1.46	1.38	0.08
Alpha	0.83	0.92	0.09	0.85	0.94	0.09
Anisotropy	0.49	0.47	0.02	0.51	0.49	0.02
Entropy	0.81	0.83	0.02	0.79	0.81	0.02
Shannon Entropy	0.48	0.67	0.19	0.53	0.51	0.02
Span	−10.88	−9.63	1.25	−10.37	−10.86	0.49
B2	0.25	0.67	0.42	0.29	0.53	0.24
B3	0.46	0.81	0.35	0.57	0.72	0.15
B4	0.24	0.66	0.42	0.25	0.61	0.36
B5	0.49	0.84	0.35	0.6	0.77	0.17
B6	0.68	0.93	0.25	0.77	0.85	0.08
B7	0.74	0.93	0.19	0.79	0.85	0.06
B8	0.75	0.93	0.18	0.8	0.85	0.05
B8A	0.76	0.94	0.18	0.81	0.86	0.05
B11	0.47	0.76	0.29	0.55	0.55	0
B12	0.38	0.53	0.15	0.44	0.37	0.07

**Table 3:** γ and β values of synthetic aperture radar (SAR) features.

	VV	VH	VV + VH	VV − VH	VH/VV	Alpha	Anisotropy	Entropy	Shannon Entropy	Span
γ	0.011	0.047	0.031	0.005	−0.04	−0.019	−0.011	−0.006	0.125	0.026
β	10	10	10	9	5	6	6	6	9	9

**Table 4:** γ and β values of optical indices.

	B2	B3	B4	B5	B6	B7	B8	B8A	B11	B12
γ	0.018	0.101	−0.161	0.077	0.081	0.059	0.062	0.061	0.236	0.035
β	10	8	10	10	10	10	10	10	10	10

**Table 5:** Accuracy analysis of Random Forest (RF) classification results of OSF, OSI and integrated OSF–OSI stack images.

	OSF		OSI		OSF–OSI	
	Lodge (GT)	Healthy (GT)	Lodge (GT)	Healthy (GT)	Lodge (GT)	Healthy (GT)
Lodge (CC)	673	133	657	73	686	60
Healthy (CC)	77	544	93	604	64	617
PA	90%	80%	88%	89%	91%	91%
UA	83%	88%	90%	87%	92%	91%
OA	85%		88%		91%	
Kappa	0.70		0.76		0.83	

GT: ground truth; CC: classification category.
